# Prospects of Topoisomerase Inhibitors as Promising Anti-Cancer Agents

**DOI:** 10.3390/ph16101456

**Published:** 2023-10-13

**Authors:** Prasanna Anjaneyulu Yakkala, Naveen Reddy Penumallu, Syed Shafi, Ahmed Kamal

**Affiliations:** 1 Department of Pharmaceutical Chemistry, School of Pharmaceutical Education and Research, Jamia Hamdard, New Delhi 110062, India; prasannayakkala@jamiahamdard.ac.in; 2 Department of Pharmacology, School of Pharmaceutical Education and Research, Jamia Hamdard, New Delhi 110062, India; naveenjnv97@gmail.com; 3 Department of Chemistry, School of Chemical and Life Sciences, Jamia Hamdard, Hamdard Nagar, New Delhi 110062, India; syedshafi@jamiahamdard.ac.in; 4 Department of Pharmacy, Birla Institute of Technology and Science (BITS) Pilani, Hyderabad Campus, Dist. Medchal, Hyderabad 500078, India; 5 Telangana State Council of Science & Technology, Environment, Forests, Science & Technology Department, Hyderabad 500004, India

**Keywords:** cancer, clinical, marketed drugs, pre-clinical, topoisomerase

## Abstract

Topoisomerases are very important enzymes that regulate DNA topology and are vital for biological actions like DNA replication, transcription, and repair. The emergence and spread of cancer has been intimately associated with topoisomerase dysregulation. Topoisomerase inhibitors have consequently become potential anti-cancer medications because of their ability to obstruct the normal function of these enzymes, which leads to DNA damage and subsequently causes cell death. This review emphasizes the importance of topoisomerase inhibitors as marketed, clinical and preclinical anti-cancer medications. In the present review, various types of topoisomerase inhibitors and their mechanisms of action have been discussed. Topoisomerase I inhibitors, which include irinotecan and topotecan, are agents that interact with the DNA-topoisomerase I complex and avert resealing of the DNA. The accretion of DNA breaks leads to the inhibition of DNA replication and cell death. On the other hand, topoisomerase II inhibitors like etoposide and teniposide, function by cleaving the DNA-topoisomerase II complex thereby effectively impeding the release of double-strand DNA breaks. Moreover, the recent advances in exploring the therapeutic efficacy, toxicity, and MDR (multidrug resistance) issues of new topoisomerase inhibitors have been reviewed in the present review.

## 1. Introduction

Topoisomerases are a crucial class of enzymes that play a pivotal role in various DNA-related processes, including DNA replication and transcription. They intricately control DNA topology, enabling the strands to unwind, separate, and rejoin seamlessly during essential cellular processes [1]. There are mainly two categories of topoisomerases (TOPO): topoisomerase I (TOPO I) and topoisomerase II (TOPO II). Further, there are three subcategories of type I topoisomerases: type IA, IB, and IC [2]. Type IA topoisomerases, also known as bacterial topoisomerase I, require a single-stranded region in the DNA to bind [3]. They cleave one of the strands in double-stranded DNA and process a transient covalent bond (*via* tyrosine residue) with the 5’-phosphoryl group of the DNA [4,5]. This covalent attachment allows the enzyme to pass the unbroken DNA strand through the nick, altering the DNA topology. The process by which the enzyme allows the DNA to pass through is referred to as the “strand passage” mechanism. Type IA topoisomerases relax negative supercoils, resolve DNA knots, and facilitate DNA replication and transcription [6].

Whereas, type IB topoisomerases are discovered in both prokaryotes and eukaryotes, including humans. This subtype includes human topoisomerase I. Type IB topoisomerases break one strand of the DNA, similar to type IA enzymes [1]. However, they differ in the attachment of the catalytic site of tyrosine residue [7]. Type IB topoisomerases covalently link the tyrosine to the 3’-phosphoryl group of the DNA [8]. They use a mechanism called the “swivel” mechanism to relax the DNA supercoils. In this mechanism, the enzyme interacts with the DNA, breaks one strand, allows the DNA to rotate around the intact strand, and finally reveals the nick [9]. Type IB topoisomerases are involved in DNA replication, transcription, repair, and chromatin remodeling. Type IC topoisomerases are structurally similar to type IB enzymes and share some functional characteristics. They break a single strand in the DNA and covalently link with the catalytic-pocket tyrosine to the 3’-phosphoryl end of the DNA [10]. Type IC topoisomerases follow the swivel mechanism to relax the DNA supercoils, similar to type IB topoisomerases. These enzymes were primarily discovered in archaea, but they are also present in some bacteria and viruses. Their specific functions and role in DNA metabolism are still being studied [11].

Similarly, TOPO II also consists of two subtypes: type IIA and IIB. It is probable that TOPO IIA plays a key function in the cell division process. TOPO IIB is expressed in post-mitotic cells and may be important in regulating the expression of long genes, even at this early stage [12].

### 1.1. Topoisomerase I Mechanism of Action

**Figure 1:**
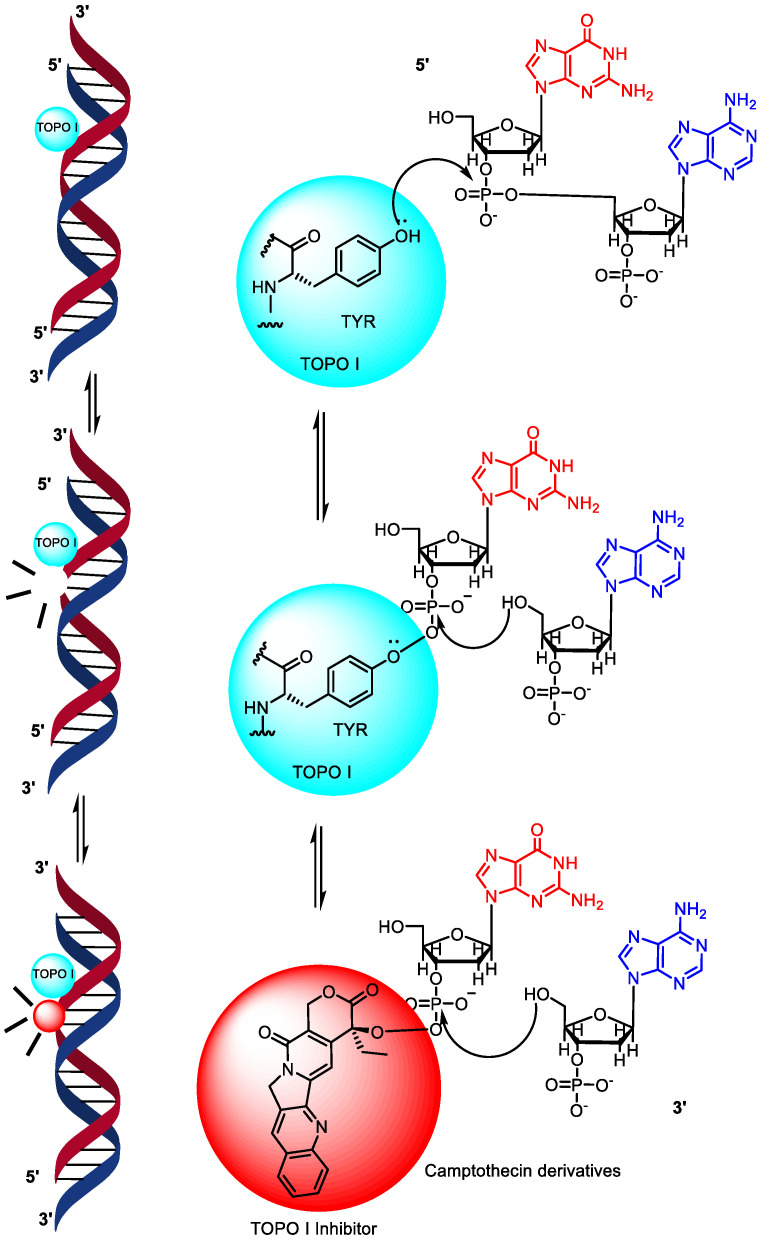
Mechanism of action of topoisomerase I.

The general mechanism of action (MOA) of TOPO I involves the relaxation of DNA supercoiling and the prevention of DNA torsional stress at the outset; TOPO I recognizes and binds to a specific DNA sequence, usually regions with single-stranded DNA, through non-covalent interactions [13]. Then, TOPO I introduces a reversible cleave in one of the DNA strands [14]. This break occurs by forming a covalent linkage between the active site or catalytic site of tyrosine (TYR) residue and the 3’-phosphoryl group of the DNA [15]. Inhibitors of TOPO I interfere with the denaturation and annealing of the DNA backbone, which is important for DNA replication and transcription. By blocking the action of TOPO enzymes, it is possible to disrupt the ligation step in DNA during replication or transcription, leading to DNA cleavage [16]. These breakdowns can disrupt the genomic integrity of cells and activate mechanisms, such as apoptosis or necrosis (Figure 1) [17].

Mammalian cells typically possess seven different active sites in topoisomerase enzymes (Figure 1), with four encoding type I enzymes and three encoding type II enzymes. Type I topoisomerases are monomeric enzymes discovered in mammalian cells [18]. They bind to double-stranded DNA and relieve torsional stress during transcription by cleaving one strand of DNA. TOPO I drugs or inhibitors are primarily employed in colorectal and ovarian cancer treatments [19]. Examples of topoisomerase I inhibitors include camptothecin derivatives, particularly camptothecin **9**, topotecan **10**, irinotecan **11**, and belotecan **12**, which are accepted by the U.S. Food and Drug Administration (FDA) for colon, lung, and ovarian cancer treatments, however, these drugs have certain limitations [20,21]. They can undergo spontaneous inactivation in the blood, requiring longer infusion times due to rapid drug reversal, and some cancer cells with increased membrane transporters may develop resistance to these drugs [11,13].

### 1.2. Mechanism of Action of Topoisomerase II

TOPO II is an enzyme that recognizes specific DNA sequences, usually regions with double-stranded DNA, during its mechanism of action (MOA) [22]. It cleaves both strands of the double helix, creating double-strand breaks in a coordinated manner [23,24]. Unlike type I topoisomerases, type II topoisomerase enzymes exist as multimeric complexes with α and β domains. Inhibitors of TOPO II induce double-strand breaks, leading to the activation of apoptosis in cells [6,25]. The capability of TOPO II in the denaturation and annealing of DNA is central to its active site. All topoisomerases utilize catalytic site tyrosine amino acid residues to facilitate DNA distraction and ligation. Type II enzymes, which break both strands of the double helix, have one of these tyrosine residues in each protomer subunit [11,26]. TOPO II starts DNA breakdown through the nucleophilic attack of the catalytic position of the tyrosine amino acid residue on the 3’ end of the phosphate group in the nucleic acid backbone [24].

**Figure 2:**
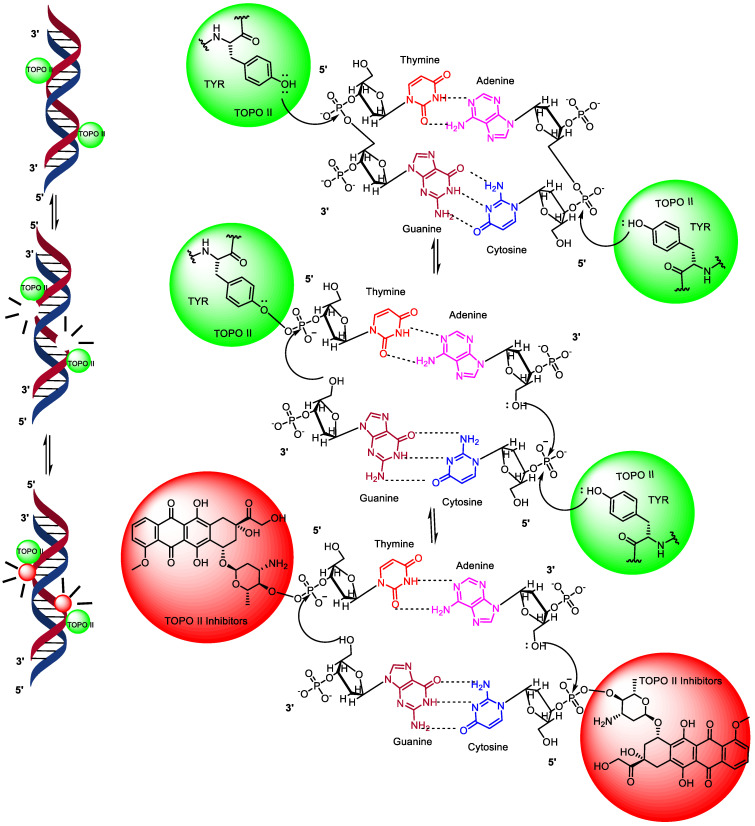
Topoisomerase II mechanism of action.

As a result, a covalent phosphotyrosine bond is formed that binds the protein to the freshly generated 5’ end of the DNA chain [27,28]. Simultaneously, a 3’-hydroxyl group is generated on the opposite end of the cleaved strand. The cleavage occurs at staggered scissile bonds across the major groove in the double helix, generating four-base 5’ single-stranded cohesive ends that are covalently linked to separate protomer subunits of the enzyme. TOPO II inhibitors used in anti-cancer drugs exert their effects by interfering with the enzyme’s mechanism of action. They induce double-strand breaks, leading to DNA damage and the activation of apoptosis in cancer cells [29]. Drugs, such as anthracycline, anthracenedione, acridine, and epipodophyllotoxin derivatives, are well-known inhibitors of topoisomerase II [10,30,31] as shown in Figure 2.

**Figure 3:**
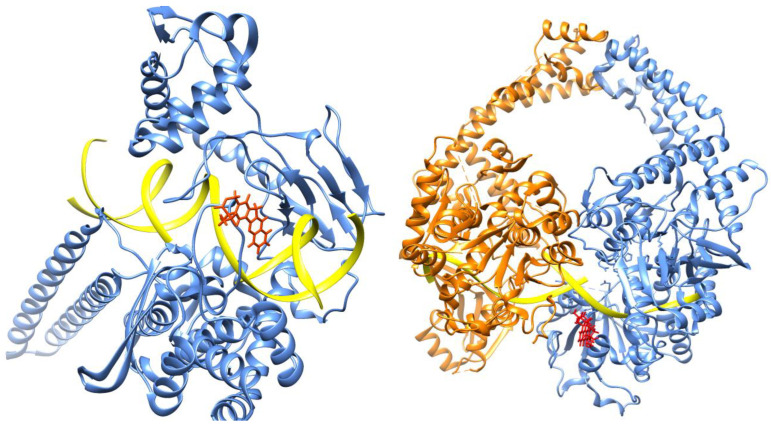
Structures of human topoisomerases. Shown are the structures of the full-length human topoisomerase I (left, PDB ID 1k4t) and topoisomerase IIβ (right, PDB ID 5gwi) enzymes with bound DNA, representative of the overall structure and domains of the two sub-family types.

In recent literature, several noteworthy reviews have been published detailing the crystal structures of topoisomerase enzymes in a complex with their corresponding inhibitors. Specifically, these reviews have shed light on the structural characteristics of both topoisomerase I (TOPO I) and topoisomerase II (TOPO II) proteins. Here, we provide a comprehensive overview of the protein structures complex with TOPO, as revealed by these crystallographic studies [1,32,33] as depicted in Figure 3.

## 2. Drugs in Clinical Usage

### 2.1. Anthracycline-Based Clinically Used DNA-Topoisomerase Inhibitors

The anthracycline agents were the primary class of TOPO inhibitors used in cancer treatment. They were initially discovered in bacterial Streptomyces species and found to have anti-proliferative and antibiotic properties [32]. Some clinically marketed anthracycline congeners are doxorubicin **1**, epirubicin **2**, valrubicin **3**, daunorubicin **4**, and idarubicin **5**. Doxorubicin, in particular, is used for various cancers, such as breast cancer, leukemia, lymphoma, sarcomas, carcinomas, and other tumors [34]. Daunorubicin and idarubicin are used for leukemia [35], while epirubicin is used after breast cancer surgery and valrubicin for urinary bladder carcinoma [36,37,38]. Anthracycline agents exert their cytotoxic effects by acting as “poisons” for topoisomerases, specifically type IIα topoisomerases (TOPO IIα and TOPO IIβ). They intercalate into DNA, bind to it, and are subsequently broken down by topoisomerases. This interaction stabilizes the DNA–TOPO complex, inhibiting DNA re-ligation [39,40].

**Figure 4:**
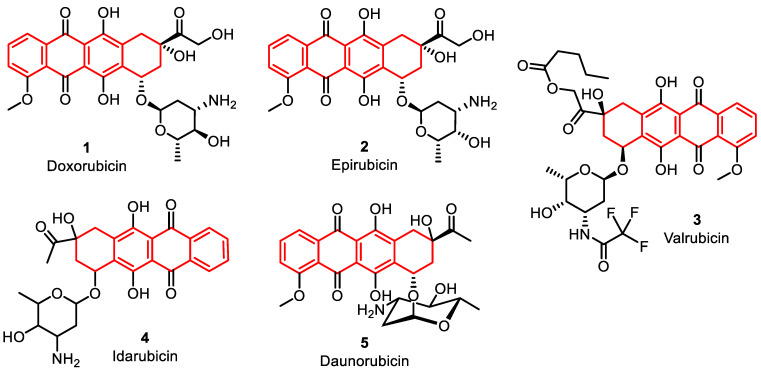
Anthracycline derivatives as DNA–topoisomerase inhibitors.

This mechanism contributes to their ability to induce cell death. Additionally, anthracyclines generate free radicals in an iron-dependent manner, which further enhances their cytotoxic effects on cancer cells [41]. Recent studies advise that the inhibition of TOPO IIβ, which is excessively expressed in cardium, by anthracycline congeners may lead to cardiotoxicity through apoptosis and reactive oxygen species (ROS) production [42] (Figure 4, Table 1).

### 2.2. Anthracenedione and Acridine Derivatives

**Figure 5:**
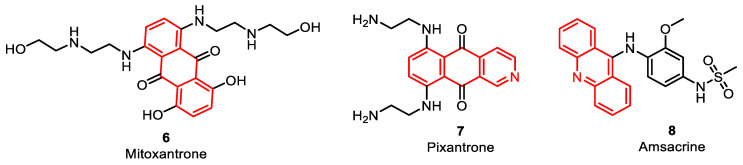
Anthracenedione and acridine analogues as DNA–TOPO inhibitors.

Anthracenedione agents, including mitoxantrone **6** and pixantrone **7**, are synthetic drugs that mimic the action of anthracycline drugs (Figure 5). Analogous to anthracyclines, anthracenedione agents act as topoisomerase poisons, primarily targeting TOPO II. Mitoxantrone intercalates into DNA bound by topoisomerase, avoiding DNA re-ligation and finally causing DNA breakage and interruption of the DNA repair processes. Pixantrone, an aza-anthracenedione, was accepted for the treatment of non-Hodgkin B-cell lymphoma. It depicts cytotoxic effects through DNA intercalation, similar to anthracyclines. Animal models have shown that treatment with doxorubicin leads to increased heart weight, whereas treatment with pixantrone does not have the same effect [43] (Table 1).

Amsacrine (m-AMSA **8**) is the only marketed agent in its chemical class. It is a synthetic drug that consists of an acridine ring (Figure 5). Similar to the previously discussed agents, amsacrine acts as a TOPO inhibitor, specifically aimed at type II TOPO. Notably, amsacrine was the first drug recognized to inhibit eukaryotic TOPO II. The acridine ring of the amsacrine is responsible for its intercalation into DNA and contributes to its activity. On the other hand, the non-interactive m-AMSA head group provides specificity for the DNA–TOPO cleavage complex. This combination of structural components enables amsacrine to exert its cytotoxic effects [44] (Table 1).

### 2.3. Camptothecin Analogues

**Figure 6:**
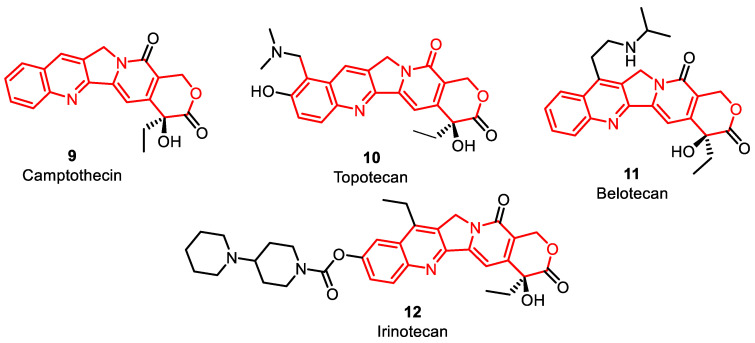
Camptothecin derivatives as DNA–topoisomerase inhibitors.

Several clinically used camptothecin derivatives are available in the market, including camptothecin **9**, topotecan **10**, irinotecan **11**, and belotecan **12** (Figure 6). These derivatives stem from the camptothecin alkaloid, initially obtained from the Chinese tree *Camptotheca acuminata*. Camptothecin-derived congeners act initially as topoisomerase inhibitors affecting TOPO I. Many researchers have demonstrated that camptothecin inhibits TOPO I, leading to DNA strand breaks and inhibition of DNA replication. Irinotecan and topotecan are clinically accessible hydrophilic versions of camptothecin. They reversibly coordinate and form a ternary complex with TOPO I and DNA, as mentioned earlier [1,41]. The FDA approved it as a second-line treatment for lung cancer, with a cisplatin **27** combination for stage IV-B cervical carcinoma patients instead of surgery or radiation [42,43]. Irinotecan is used for metastatic colon or rectal carcinoma following treatment failure or progression with fluorouracil **26** [45,46,47,48]. It can also be given in combination with leucovorin **28** and 5-fluorouracil [49] (Figure 9). Belotecan, one of the comparatively newer camptothecin congeners, has been approved in South Korea for lung and ovarian cancer treatment since 2003. It shares the same MOA as other agents in its class. In comparison to previously known camptothecin-based agents, belotecan demonstrates similar efficacy with decreased toxicities [50] (Figure 6, Table 1).

### 2.4. Epipodophyllotoxin Derivatives

**Figure 7:**
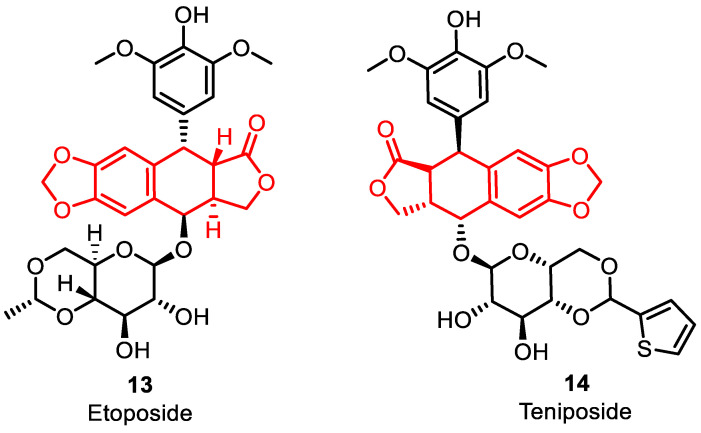
Epipodophyllotoxin derivatives as DNA–topoisomerase inhibitors.

Etoposide **13** and teniposide **14** (Figure 7) were obtained from epipodophyllotoxins, which are natural sources obtained from the mayapple plant (*Podophyllum peltatum*). These drugs act as topoisomerase poisons by binding to type II TOPO, similar to the derivatives discussed earlier. Etoposide has been used in combination with chemotherapy regimens for refractory testicular tumors and as part of the treatment for lung cancer in combination with cisplatin [51]. Teniposide is approved for use in combination with other chemotherapy medications to treat refractory pediatric acute lymphoblastic leukemia [52].

**Table 1:** Marketed topoisomerase inhibitors.

Sl.	Drug	Class	Mechanism/Target	Indications	Adverse Drug Reactions	Ref.
1	Doxorubicin	Anthracycline	Topoisomerase IIα and IIβ poison, intercalation, free radicals	Various cancers	Cardiotoxicity, myelosuppression, nausea, potential for cumulative toxicity	[53,54]
2	Epirubicin	Anthracycline	Topoisomerase IIα and IIβ poison, intercalation, free radicals	Breast cancer	Cardiotoxicity, myelosuppression, nausea, potential for cumulative toxicity	[36]
3	Valrubicin	Anthracycline	Topoisomerase IIα and IIβ poison, intercalation, free radicals	Urinary bladder carcinoma	Local irritation, urinary symptoms, myelosuppression	[55]
4	Daunorubicin	Anthracycline	Topoisomerase IIα and IIβ poison, intercalation, free radicals	Leukemia	Cardiotoxicity, myelosuppression, nausea, potential for cumulative toxicity	[56]
5	Idarubicin	Anthracycline	Topoisomerase IIα and IIβ poison, intercalation, free radicals	Leukemia	Cardiotoxicity, myelosuppression, nausea, potential for cumulative toxicity	[57]
6	Mitoxantrone	Anthracenedione	Topoisomerase II poison, intercalation	Leukemia, prostate cancer, MS	Myelosuppression, potential for cumulative toxicity, potential for myelosuppression	[58]
7	Pixantrone	Anthracenedione	Topoisomerase II poison, intercalation	Non-Hodgkin B-cell lymphoma	Myelosuppression, potential for cumulative toxicity	[43]
8	Amsacrine	Acridine	Topoisomerase II poison, intercalation	Acute leukemia	Myelosuppression, potential for cumulative toxicity	
9	Camptothecin	Camptothecin	Topoisomerase I inhibitor, DNA strand breaks	Not specified	Gastrointestinal toxicity, myelosuppression, potential for cumulative toxicity	[59]
10	Topotecan	Camptothecin	Topoisomerase I inhibitor, DNA strand breaks	Small-cell lung cancer	Myelosuppression, gastrointestinal toxicity, potential for cumulative toxicity	[60,61]
11	Irinotecan	Camptothecin	Topoisomerase I inhibitor, DNA strand breaks	Colon and rectal carcinoma	Diarrhea, myelosuppression, potential for cumulative toxicity	[62]
12	Belotecan	Camptothecin	Topoisomerase I inhibitor, DNA strand breaks	Non-small-cell lung cancer, ovarian cancer	Myelosuppression, gastrointestinal toxicity, potential for cumulative toxicity	[50]
13	Etoposide	Epipodophyllotoxin	Topoisomerase II poison, intercalation	Testicular tumors, small-cell lung cancer	Myelosuppression, gastrointestinal toxicity, potential for cumulative toxicity	[63]
14	Teniposide	Epipodophyllotoxin	Topoisomerase II poison, intercalation	Childhood acute lymphoblastic leukemia	Myelosuppression, gastrointestinal toxicity, potential for cumulative toxicity	[64]

## 3. TOPO Inhibitors in Clinical Trials

This segment is focused on novel and new TOPO inhibitor scaffolds that were examined in human clinical trials, with a summary provided in Table 2. The clinical trials discussed in this section are categorized based on their phase (1, 2, or 3) and include relevant identifiers, such as National Clinical Trial (NCT) numbers or other regulatory agency identifiers. The information is sourced from the U.S. National Library of Medicine’s clinical trials database and the WHO/ICMJE ISRCTN registry.

### 3.1. Topoisomerase Inhibitors in Phase 1 Clinical Trials

**Figure 8:**
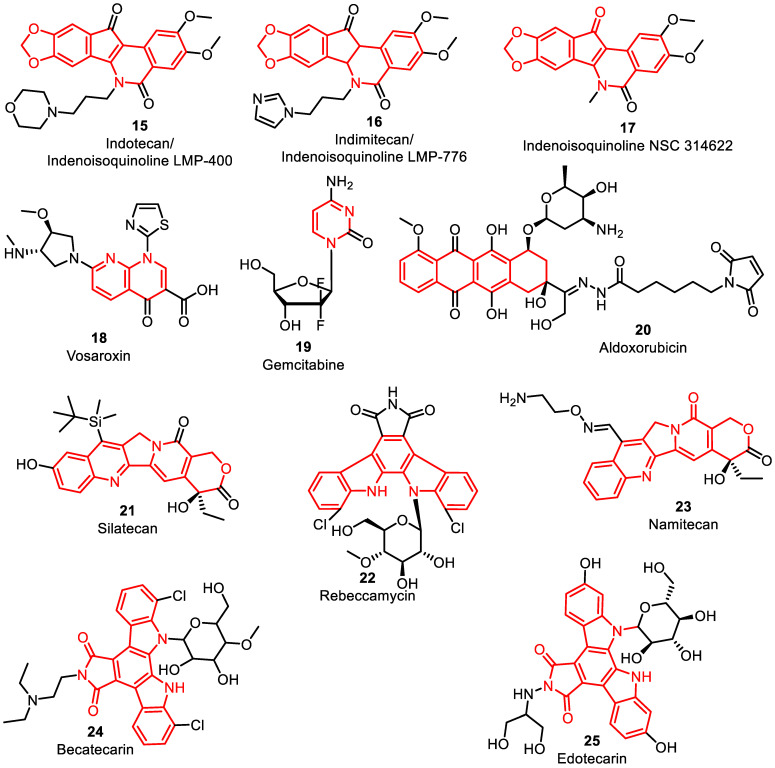
Representative DNA–TOPO inhibitors in clinical trials.

The U.S. National Cancer Institute (NCI) conducted a phase 1 clinical trial (NCT-01794104, (Figure 8, Table 2) investigating a novel class of non-camptothecin type I topoisomerase poisons, called indenoisoquinolines, indotecan **15,** inimitecan **16,** and indenoisoquinoline NSC 314622 **17,** for neoplasm lymphoma [65]. Indenoisoquinolines create a stable DNA–TOPO cleavage complex like camptothecin analogues, but exhibit a preference for specific DNA cleavage sites. This preference enables them to effectively target camptothecin-resistant cell lines. These derivatives are chemically stable and target their action on cells that overexpress ATP-binding cassette transporters ABCG2 and P-glycoprotein (MDR1). By stabilizing the breakdown complex, they induce DNA destruction, representing their efficacy as potent anti-cancer treatments. Furthermore, indenoisoquinolines delay DNA repair, leading to apoptosis. After five days of administration, LMP400 has linear pharmacokinetics, at which point drug aggregation is seen. Weekly dosing is thought to raise the drug’s peak levels, resulting in improvements in safety and efficacy [66].

Namitecan (ST1968) **23** is a TOPO I inhibitor that has demonstrated good anti-proliferative activity and has a far better safety profile compared to topotecan **10** and irinotecan **12**. Pharmacokinetic studies using repeated dosing schedules have shown no production or accumulation of metabolites due to its short half-life. Current research has validated the safety and pharmacokinetic profile of namitecan, including manageable neutropenia, and has shown efficacious anti-proliferative activity, with positive responses observed in endometrium and bladder cancers [67].

### 3.2. Topoisomerase Inhibitors in Phase 2 Clinical Trials

**Figure 9:**
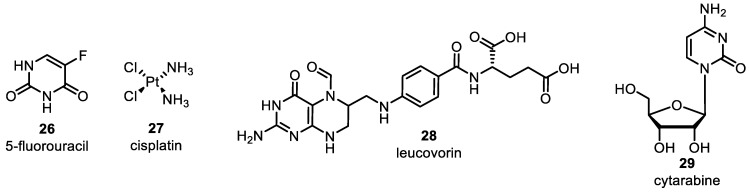
Miscellaneous DNA–topoisomerase inhibitors.

The Vanderbilt-Ingram Cancer Center enrolled subjects for phase 2 clinical trials (NCT-02658487) to evaluate the effectiveness of vosaroxin **18** and cytarabine **29** (Figure 9) in treating patients with untreated acute myeloid leukemia. Vosaroxin is an anti-proliferative quinolone conjugate that targets type II TOPO (Figure 8). Quinolone conjugates act on the DNA–TOPO to breakdown complex and intercalate DNA at exact GC-rich sites to avoid DNA annealing by TOPO. It leads to specific site DNA destruction, prolongation of the S-phase, G2-phase cell cycle arrest, and finally leads to apoptosis [32]. Vosaroxin consists of a quinolone core, which makes it less active compared to the remaining classes of TOPO inhibitors. It produces fewer toxic metabolites, ROS and cardiotoxicity. Furthermore, vosaroxin can induce p53-independent apoptosis, making it effective against drug resistance mechanisms associated with p53 inactivation. Its stable quinolone structure is not extensively metabolized by enzymes or capable of inducing or inhibiting p450 activity, minimizing the potential for drug–drug interactions and even enhancing the activity of other anti-cancer drugs, such as cytarabine [68].

The primary goal of this research is to assess the proportion of patients achieving complete remission after undergoing initial treatment with a combination of vosaroxin and cytarabine. This study focuses on individuals who have recently been diagnosed with acute myelogenous leukemia and have not received any prior treatment. The aim is to evaluate the effectiveness of the vosaroxin and cytarabine combination during the induction therapy phase [69]. In another phase 2 clinical trial completed by NewLink Genetics Corporation (NCT-01380769), the impact of CRLX101 on the average survival of patients with advanced non-small-cell lung cancer (NSCLC) was studied. CRLX101 is a camptothecin nanoparticle conjugated to a cyclodextrin-based polymer, designed to increase tumor cell exposure to camptothecin, while minimizing side effects. The nanoparticle size facilitates tumor-specific targeting by extravasating from the leakier blood vessels found in tumors [70] (Figure 8).

### 3.3. Topoisomerase Inhibitors in Phase 3 Clinical Trials

CTI BioPharma performed a phase 3 study (NCT-01321541) comparing the ability of pixantrone **7** with rituximab to gemcitabine **19** with rituximab in 260 patients with relapsed or refractory diffuse large B-cell lymphoma or follicular grade 3 lymphoma. The major results measure in the study was progression-free survival (PFS), with secondary outcome measures including total survival, and complete and total response rate, as well as safety evaluations, such as adverse events and laboratory values falling outside predetermined ranges [71]. Aldoxorubicin **20**, a pro-drug of doxorubicin, is considered a promising option for the treatment of soft tissue sarcomas [72]. It contains a carboxylic hydrazine that binds to albumin in the blood and is then released in the acidic tumor environment, delivering doxorubicin directly to the tissue. A phase 3 study, sponsored by CytRx (NCT-02049905), investigated the administration of aldoxorubicin to patients with soft tissue sarcomas, with the active comparator being the investigator’s choice among various treatment options. The study assessed the overall survival, safety parameters, and tumor response. Other notable topoisomerase inhibitors undergoing clinical trials include silatecan **21**, a silicon-containing camptothecin analogue [73], and rebeccamycin analogs obtained from the natural product rebeccamycin **22 [74]**. Silatecan is being evaluated in a phase 2 study for gliosarcoma (NCT-01124539), while rebeccamycin analogs, such as becatecarin **24** and edotecarin **25**, have progressed to phase 2 trials. These compounds exhibit dual TOPO I and II inhibitors. However, the clinical development of becatecarin and edotecarin has stopped, and it remains uncertain whether additional rebeccamycin compounds will be tested clinically (Figure 8) [75].

## 4. Topoisomerase Inhibitors in Preclinical Studies

### 4.1. Naphthalimide–Benzothiazole Derivatives

**Figure 10:**
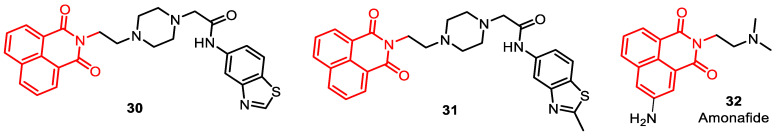
Naphthalimide–benzothiazole derivatives DNA–topoisomerase inhibitors.

Rao et al. reported novel naphthalimide–benzothiazole derivatives as topoisomerase IIα inhibitors. Among the series of compounds, compounds **30** and **31**, containing the 6-aminobenzothiazole ring, exhibited significant cytotoxic activity against lung cancer (IC_50_: 4.074 and 3.890 μM) and colon cancer (IC_50_: 3.715 and 3.467 μM) cell lines when compared to the standard compound (amonafide **32**) (IC_50_: 5.459 and 7.762 μM). They also investigated the DNA-binding properties of the active analogues using various techniques, such as DNA viscosity, CD, UV/Vis, fluorescence spectroscopy, and molecular docking, revealing a strong intercalation between the two DNA strands. Additionally, the most potent analogues, **30** and **31**, were successfully inhibited by DNA TOPO II (Figure 10) [76].

### 4.2. β-Carboline Hybrids as Topoisomerase Inhibitors

**Figure 11:**
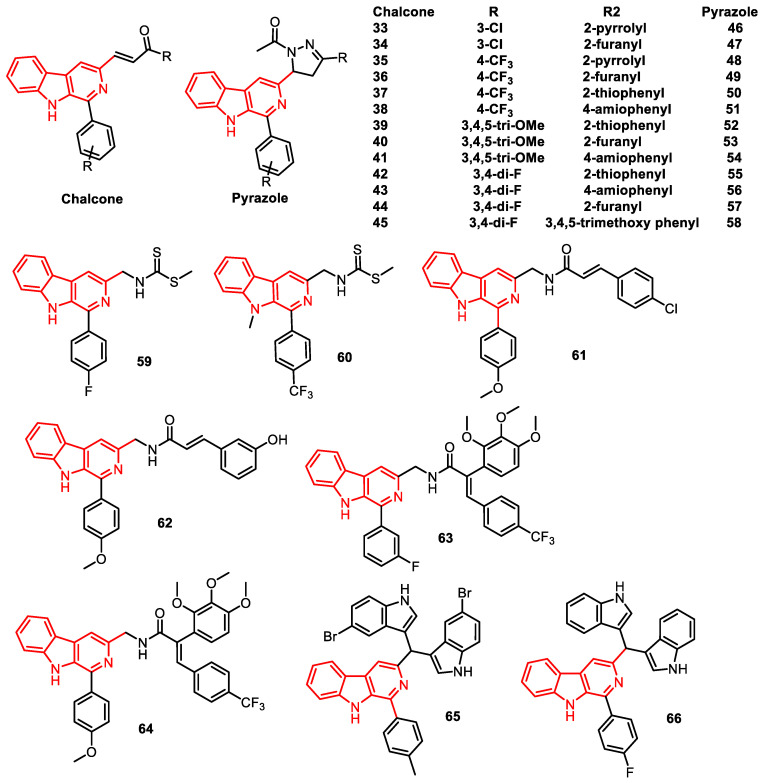
β-Carboline analogues as DNA–topoisomerase inhibitors.

In our previous studies, we have described the topoisomerase I inhibitory potential of β-carboline hybrids (**33**–**58**). A series of new β-carboline hybrids were prepared by introducing a phenyl group at the C1 position, along with chalcone/(N-acetyl)/pyrazole molecules at the position C3, and all the synthesized analogues were assessed for their anti-proliferative activity. Additionally, DNA photocleavage studies demonstrated that two of the analogues, compound **36** and compound **49**, successfully cleaved pBR322 plasmid DNA upon UV light irradiation. The potent hybrid compound **49** efficiently inhibited DNA TOPO I activity and maintained the DNA in the supercoiled form (Figure 11) [77].

In another study, dithiocarbamate-linked β-carboline analogues were reported as DNA TOPO II inhibitors. Among the derivatives, compounds **59** and **60** displayed a prominent anti-proliferative profile, with IC_50_ values of 1.34 µM and 0.79 µM on the DU-145 cell line, respectively. Both biophysical investigations and in silico studies indicated a complexion-type interaction between these analogues and DNA, distinguishing them from simple β-carbolines. To gain insight into their MOA, a DNA TOPO II inhibition assay was also performed (Figure 11) [78].

Similarly, C3-trans-cinnamide linked β-carboline analogues were described as significant anti-proliferative and DNA TOPO I poisons. These analogues were subjected to the cytotoxic profile against selected human cancer cell lines. The results indicated that the newly designed analogues displayed prominent activity against all the tested cell lines, having IC_50_ values in the range of 13–45 nM. Particularly, conjugates **61** and **62** demonstrated the highest activity against the breast cancer cell line (MCF-7 cells), with IC_50_ values of 14.05 nM and 13.84 nM, respectively. They also investigated the TOPO I inhibition assay, DNA-binding affinity, and in silico studies, disclosing that these novel analogues function as DNA-interactive TOPO I inhibitors (Figure 11) [79].

In related studies, β-carboline-combretastatin carboxamides were reported as DNA intercalation and TOPO II inhibitors. They were subjected to such studies for their potential DNA-binding affinity, cytotoxicity, and TOPO II inhibition activity. Among them, compounds **63** and **64** demonstrated potent cytotoxic effects against the A549 cell line, with IC_50_ values of 1.01 µM and 1.17 µM, respectively. The most potent conjugate, **63**, was tested for DNA TOPO II inhibition activity (Figure 11) [80].

In another example, β-carboline-*bis*indole analogues were described as DNA binding, photocleavage agents, and TOPO I inhibitors. A series of β-carboline-*bis*indole analogues were synthesized and subjected to studies for their anti-proliferative activity against various human cancer cell lines. All the analogues demonstrated significant anti-proliferative activity. Particularly, compounds **65** and **66** exhibited noteworthy activity against DU-145 cells, with IC_50_ values of 1.86 µM and 1.80 µM, respectively. The active conjugates significantly inhibited DNA TOPO I enzyme and were capable of cleaving the pBR322 plasmid under UV light irradiation. The results also reported a combilexin-type interaction between the compounds and DNA (Figure 11) [81].

### 4.3. Imidazopyridinyl-1,3,4-Oxadiazole Derivatives

**Figure 12:**
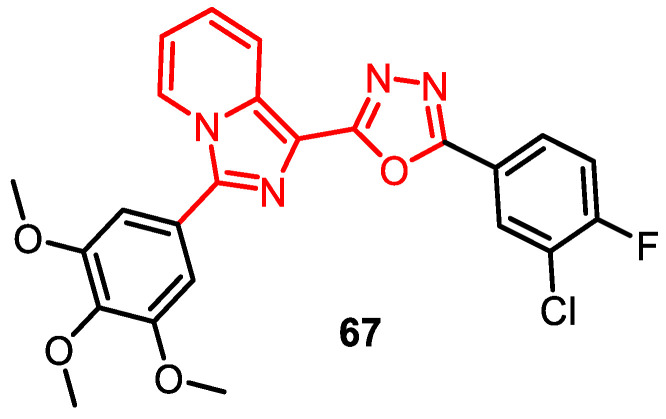
Imidazopyridinyl-1,3,4-oxadiazole derivatives as DNA–TOPO inhibitors.

A series of imidazopyridinyl-1,3,4-oxadiazole analogues were synthesized and subjected to an anti-proliferative assay, revealing promising results for some compounds. Notably, compound **67** (NSC 763639) exhibited significant growth inhibition with a single dose (10 µM) across all human cancer cell lines, meeting the threshold criteria. This compound was used in five different dose levels (0.01, 0.1, 1, 10, and 100 µM) and yielded GI_50_ values ranging between 1.30 to 5.64 µM. Conjugate **67** exhibited significant inhibition of TOPO II activity, as observed in the TOPO II-mediated DNA relaxation assay (Figure 12) [82].

### 4.4. Pyrazole-Linked Benzothiazole-β-Naphthol Derivatives

**Figure 13:**
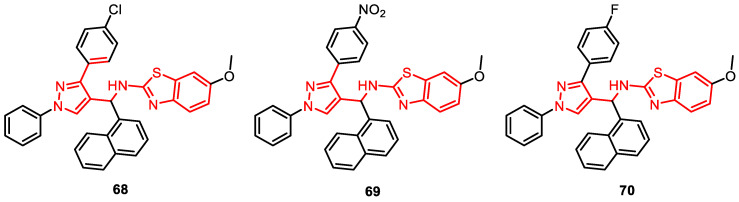
Pyrazole-linked benzothiazole-β-naphthol analogues as DNA–topoisomerase inhibitors.

Pyrazole-linked benzothiazole-β-naphthol analogues were synthesized using environmentally friendly methods without the need for catalysts, yielding good to excellent yields. Notably, some derivatives, **68**, **69**, and **70**, exhibited prominent cytotoxicity against human cervical cancer cells (HeLa), with IC_50_ values ranging from 4.63–5.54 µM. Furthermore, these derivatives effectively inhibited TOPO I activity (Figure 13) [83].

### 4.5. Podophyllotoxin Congeners 

**Figure 14:**
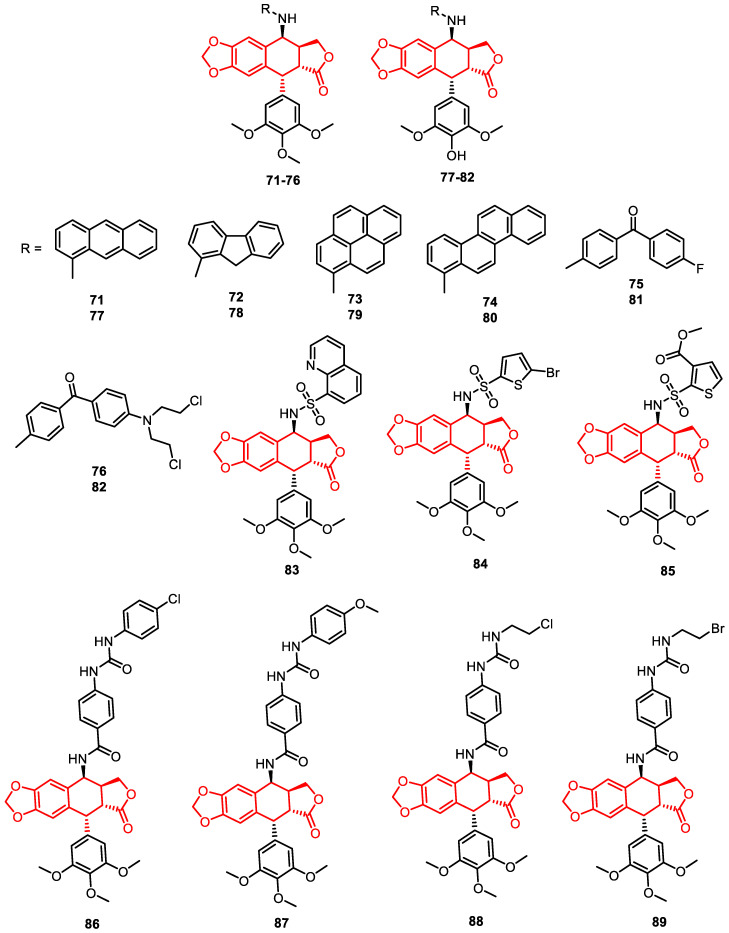
Podophyllotoxin congeners as DNA–topoisomerase inhibitors.

Kamal and others reported a novel class of polyaromatic podophyllotoxin (**71**–**82**) congeners, specifically 4β-N-polyaromatic substituted podophyllotoxins, as DNA TOPO inhibitors (Figure 14) [84].

Similarly, novel 4β-sulphonamido and 4β-[(4-sulphonamido)benzamide] podophyllotoxin analogues demonstrated potential DNA topoisomerase IIα inhibitory potential. Among them, compounds **83**, **84**, and **85** exhibited greater potency than etoposide, a known anti-cancer drug. Additionally, compound **84** triggered both single-strand and double-strand DNA breaks, as noticed through a comet assay and c-H2AX analysis, respectively. Western blot analysis and related studies confirmed that compound **84** inhibited TOPO IIα activity. The compounds also activated ATM and Chk1 proteins, indicating effective DNA damage. Moreover, compound **84** induced apoptotic cell death, as evidenced by the triggering of caspase-3, the upregulation of p21 and p16, the downregulation of NF-kB, and the decreased expression of the Bcl-2 protein (Figure 14) [85].

In another study, 4β-[4′-(1-(aryl)ureido)benzamide]podophyllotoxin congeners were reported as DNA TOPO I and IIα inhibitors. Some conjugates, **86**, **87**, **88**, and **89**, exhibited significant anti-proliferative activity in Colo 205 cells, surpassing the effectiveness of etoposide. Furthermore, enhanced inhibitory activities against DNA TOPO I and IIα enzymes were demonstrated. The active analogues also induced apoptosis by upregulating the caspase-3 protein, as observed through Western blotting and ELISA analysis (Figure 14) [86].

**Figure 15:**
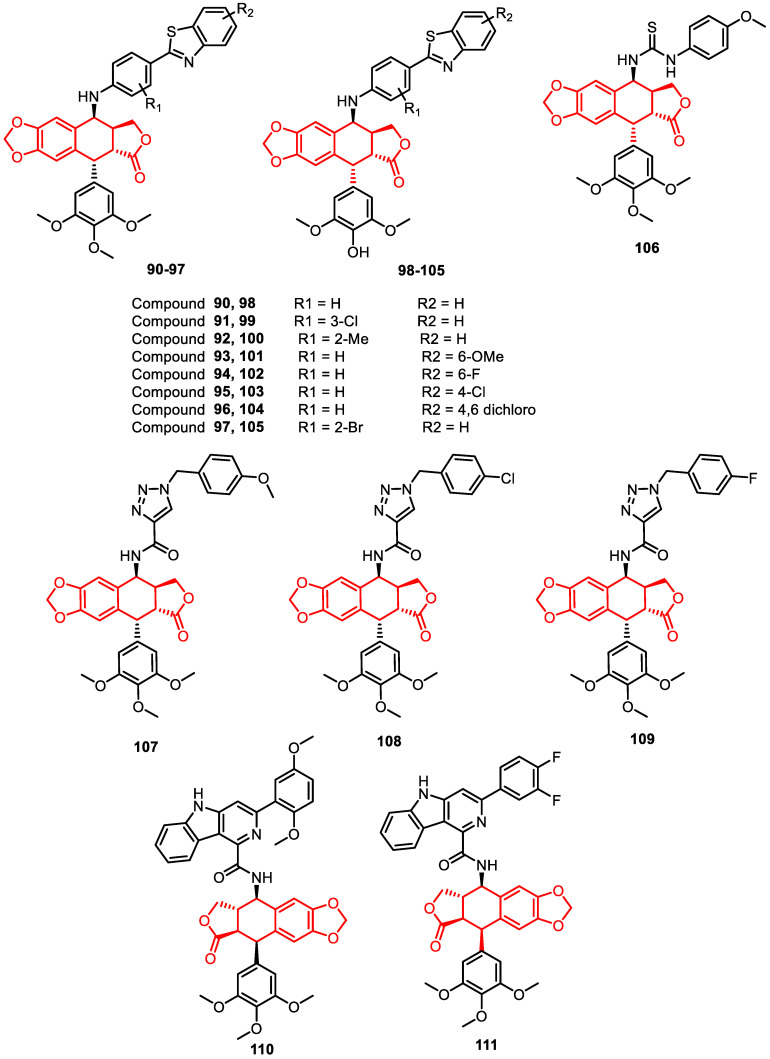
Podophyllotoxin derivatives as DNA–topoisomerase inhibitors.

A highly efficient one-pot iodination method, utilizing zirconium tetrachloride and sodium iodide, has been successfully developed for synthesizing benzothiazolo-4β-anilino-podophyllotoxin (**90**–**97**) and benzothiazolo-4β-anilino-4-O-demethylepipodophyllotoxin (**98**–**105**) analogues. Selected representative conjugates were assessed with an anti-proliferative assay against specific human cancer cell lines and their capability to inhibit DNA TOPO II activity (Figure 15) [87].

Similarly, podophyllotoxin–thiourea congeners were reported as DNA TOPO II inhibitors. Among them, selective cytotoxicity was observed on DU-145 (human prostate cancer) cells, with compound **106** displaying the most potent activity (IC_50_ of 0.50 ± 0.03 μM). Importantly, it demonstrated a favorable safety therapeutic window, exhibiting a 4-fold difference in potency compared to the non-cancerous human prostate cell line (RWPE-1, IC_50_ of 40.85 ± 0.78 μM). Flow cytometric analysis of this compound (**106**) revealed a significant G2/M-phase arrest and notable inhibition of TOPO II activity (Figure 15) [88].

In another study, a series of novel 4β-amidotriazole-linked podophyllotoxin congeners were designed and synthesized using click chemistry and assessed for their biological activities. Notably, compounds **107**, **108**, and **109** displayed remarkable cytotoxicity, with IC_50_ values of less than 1 µM against the tested cancer cell lines, surpassing the activity of the reference etoposide. Furthermore, these derivatives efficiently inhibited the activity of TOPO II, as demonstrated by topoisomerase-mediated DNA relaxation assays (Figure 15) [89].

Similarly, podophyllotoxin-linked β-carbolines analogous were reported as a significant anti-cancer agent and DNA TOPO II inhibitor. Among them, compounds **110** and **111** exhibited the highest cytotoxicity against the DU-145 cell line, with IC_50_ values of 1.07 ± 0.07 µM and 1.14 ± 0.16 µM, respectively. They also investigated cell cycle analysis, DNA-binding studies, a comet assay, TOPO II inhibition, and molecular modelling, which revealed that these derivatives interact with DNA and function as inhibitors of topoisomerase II (Figure 15) [90].

### 4.6. Benzimidazoles Congeners

**Figure 16:**
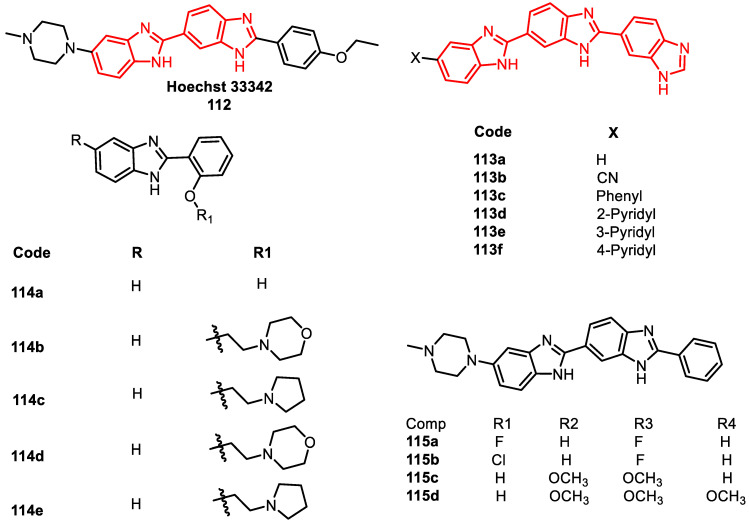
Benzimidazole derivatives as DNA–topoisomerase inhibitors.

Benzimidazoles have demonstrated their ability to disrupt DNA topoisomerases, as the significant class of enzymes involved in DNA manipulation. Notably, bibenzimidazole and terbenzimidazole compounds have emerged as distinctive Top1 poisons, constituting a class that stems from structural modifications of Hoechst 33342 (**112**), a blue fluorescent dye employed in DNA staining for molecular biology applications (Figure 16) [91].

The LaVoie Laboratory has been instrumental in advancing the understanding of terbenzimidazoles’ impact on DNA. Multiple terbenzimidazoles were identified to induce DNA breakdowns in the presence of mammalian TOPO I. These conjugates were also subjected to analysis for their cytotoxic effects on various human cancer cell lines, supporting the notion of TOPO I-mediated DNA cleavage. Comparisons with the TOPO I poison compound **112,** based on Hoechst 33342, highlighted the comparable potency of several terbenzimidazoles. Compounds **113a** and **113b** displayed analogous TOPO I inhibition to that of compound **112** (Figure 15). The 5-phenyl-substituted terbenzimidazole **113c** exhibited approximately half the potency of compound **112** as a TOPO I poison. Meanwhile, the 3- and 4-pyridyl analogs **113e** and **113f** demonstrated greater activity than the 2-pyridyl derivative **113d**, both as TOPO I poisons and anti-proliferative agents (Figure 16) [92].

In 2009, Coban et al. [93] conducted research involving congeners of benzimidazole with alteration mainly at the second and fifth positions. Remarkably high activity was observed with analogs **114a** and **114c** (95.4% and 90.2% supercoiled vs. relaxed DNA bands), showing significant TOPO 1 inhibition and cytotoxicity (Figure 15). Additionally, in 2011, a series of benzimidazole congeners (2-aryl-5-substituted-2,5-bisbenzimidazole) were synthesized and subjected to analysis for their capacity to induce DNA cleavage in the presence of TOPO I (**115a**–**d**). These derivatives exhibited significant cancer growth inhibition against tested cancer cell lines, with IC_50_ values falling within the micromolar range (Figure 16) [94,95].

**Figure 17:**
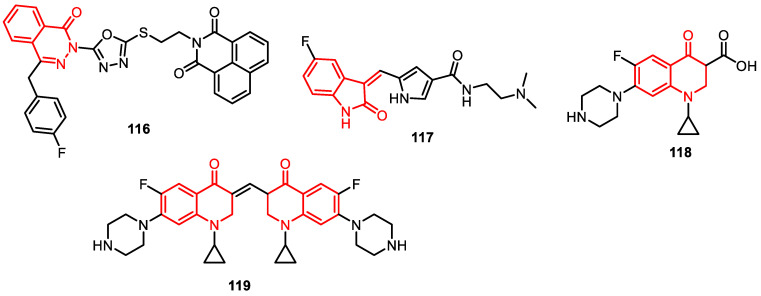
Miscellaneous derivatives as DNA–topoisomerase inhibitors.

Ananda and others developed and tested a library of 11 dual inhibitors (DiPT-1 to DiPT-11) targeting PARP1 and TOP1. DiPT-4 (**116**) emerged as a standout, effectively inducing DNA damage, cell cycle arrest, and apoptosis, while sparing normal cells. DiPT-4 stabilizes the TOPO I-DNA complex, intensifying DNA breaks and impeding PARylation, countering resistance. It is a promising candidate for improved single-agent therapy, circumventing toxicities and enhancing clinical efficacy (Figure 17) [96].

Chauhan et al. reported on fluoroquinolones, known as bacterial topoisomerase inhibitors and vital antibacterial agents, which also exhibit anti-proliferative effects attributed to their impact on eukaryotic TOPO II, inducing DNA damage similar to TOPO II poisons [97]. Additional mechanisms, like tumor growth factor regulation, might contribute to their anti-proliferative actions. Leveraging the structures of tyrosine kinase inhibitor sunitinib (**117**) and bacterial topoisomerase inhibitor ciprofloxacin (**118**) [98], researchers designed hybrid molecules (e.g., HMNE3, **119**), integrating features of both drugs. HMNE3 displayed notable inhibitory activity against tyrosine kinases and TOPO II, demonstrating cytotoxicity across multiple cell lines (Panc-1, T24, BGC-823, PU145, HCG-27, Capan-1), with nanomolar IC_50_ values [99]. Since TOPO II and the epidermal growth factor receptor (EGFR) mutually influence expression, targeting them simultaneously emerges as a promising anti-cancer approach. Novel dual inhibitors of TOPO II and EGFR have been developed to enhance treatment efficacy (Figure 17) [100].

## 5. Conclusions

In conclusion, topoisomerase inhibitors have emerged as potential anti-cancer medications due to their ability to disrupt the normal function of these enzymes and induce DNA damage, leading to cell death. This review has discussed different types of TOPO inhibitors, including type I and II inhibitors, and their mechanisms of action in the treatment of cancer. TOPO I inhibitors stabilize the cleavage complex, while TOPO II inhibitors cleave the complex with DNA, resulting in DNA breaks. These inhibitors have shown therapeutic efficacy in the treatment of various cancers, such as breast, lung, and ovarian cancer. The development of targeted therapies that selectively inhibit overexpressed isoforms of topoisomerases in cancer cells holds great promise. By specifically targeting these isoforms, the inhibitors can disrupt DNA replication and repair processes in cancer cells, while minimizing damage to normal cells. Furthermore, combining TOPO inhibitors with other anti-cancer agents, such as chemotherapeutic drugs, immunotherapies, or targeted therapies, can enhance treatment outcomes and overcome drug resistance.

Researchers have also explored natural products and structural modifications to discover and optimize new topoisomerase inhibitors. Additionally, advances in structural biology, computational modelling, and virtual screening techniques have facilitated structure-based drug design, leading to the development of inhibitors with improved drug-like properties and the ability to overcome drug resistance mechanisms. These recent advances in the discovery and design of topoisomerase inhibitors offer promising avenues to address drug resistance, enhance treatment outcomes, and progress the overall efficacy and safety of cancer therapies. New methods to improve the therapeutic efficiency include nano-delivery systems and microneedle patches. Continued research and development in this field holds great potential for advancing cancer treatment approaches.

## 6. Recent Advances in the Discovery of New and Novel Topoisomerase Inhibitors

Researchers have been working on developing targeted therapies that specifically inhibit the isoforms of topoisomerases overexpressed in cancer cells. By selectively targeting these isoforms, the inhibitors can effectively disrupt DNA replication and repair processes in cancer cells, while minimizing damage to normal cells. Combining topoisomerase inhibitors with other anti-cancer agents has shown promise in overcoming drug resistance and enhancing treatment outcomes. Combinations with chemotherapy drugs, immunotherapies, or targeted therapies can synergistically enhance the cytotoxic effects and overcome resistance mechanisms. Dual inhibitors simultaneously target multiple isoforms of topoisomerases or combine topoisomerase inhibition with the inhibition of other essential cellular processes. This approach enhances therapeutic efficacy and minimizes the likelihood of resistance development. Natural products, such as marine-derived compounds and plant extracts, continue to be a valuable source for the discovery of new topoisomerase inhibitors. Structural modifications and synthesis of analogues derived from these natural compounds can enhance their potency, selectivity, and pharmacokinetic properties. Researchers have been exploring novel DNA binding modes for topoisomerase inhibitors to enhance their affinity and specificity. By targeting unique DNA-binding sites or inducing structural changes in DNA, these inhibitors can disrupt topoisomerase function more effectively. Advances in structural biology, computational modeling, and virtual screening techniques have enabled the rational design of topoisomerase inhibitors with improved drug-like properties. By utilizing the three-dimensional structures of topoisomerases, researchers can design inhibitors with optimized binding interactions and reduced off-target effects. Understanding the molecular mechanisms of drug resistance has paved the way for the development of inhibitors that can overcome these resistance mechanisms. Strategies include the development of inhibitors that are not susceptible to drug efflux pumps, bypass resistant mutations, or target alternative DNA repair pathways. These recent advances in the discovery of topoisomerase inhibitors offer promising avenues to address drug resistance, enhance treatment outcomes, and improve the overall efficacy and safety of cancer therapies.

## Figures and Tables

**Table 2:** Clinical trials on DNA–topoisomerase inhibitors (clinicaltrials.gov, accessed on 17 August 2023).

Sl.	Drug	Class	Mechanism/Target	Study Phase	NCT Number
1	Indenoisoquinolines 15, 16, 17	Non-camptothecin type I inhibitors	Stabilize DNA–topoisomerase cleavage complex, preferential DNA cleavage sites	Phase 1	NCT-01794104
2	Namitecan (ST1968)	Topoisomerase I inhibitor	Inhibits topoisomerase I, demonstrated anti-tumor activity	Phase 1	Not specified
3	Vosaroxin	Anti-cancer quinolone derivative (AQD)	Targets type II topoisomerases, induces DNA damage and apoptosis	Phase 2	NCT-02658487
4	Cytarabine	Nucleoside analogue	Incorporates into DNA, inhibits DNA synthesis	Phase 2	NCT-02658487
5	CRLX101	Camptothecin nanoparticle conjugate	Increases tumor cell exposure to camptothecin, tumor-specific targeting	Phase 2	NCT-01380769
6	Pixantrone	Anthracenedione	Induces DNA damage, anti-tumor activity	Phase 3	NCT-01321541
7	Aldoxorubicin	Pro-drug of doxorubicin	Delivers doxorubicin directly to tumor tissue	Phase 3	NCT-02049905
8	Silatecan	Silicon-containing camptothecin derivative	Inhibits topoisomerase I, being evaluated for gliosarcoma	Phase 2	NCT-01124539
9	Becatecarin	Rebeccamycin analogue	Dual topoisomerase I and II poison, clinical development ceased	Phase 2	NCT-00132600
10	Edotecarin	Rebeccamycin analogue	Dual topoisomerase I and II poison, clinical development ceased	Phase 2	NCT-02310763

## Data Availability

Data sharing is not applicable.

## Abbreviations

ATP: adenosine triphosphate; ADP: adenosine diphosphate; TYR: tyrosine; AQD: anti-cancer quinolone derivative; DNA: deoxyribonucleic acid; NCI: National Cancer Institute; NCT: national clinical trial; PD: pharmacodynamics; TOPO I: topoisomerase 1; TOPO II: topoisomerase II; MTD: maximum tolerated dose; SAR: structure–activity relationships; PFS: progression free survival.
